# The DNA Repair Enzyme Apurinic/Apyrimidinic Endonuclease (Apex Nuclease) 2 Has the Potential to Protect against Down-Regulation of Chondrocyte Activity in Osteoarthritis

**DOI:** 10.3390/ijms150914921

**Published:** 2014-08-25

**Authors:** Naoko Yui, Hirotaka Yoshioka, Hiroto Fujiya, Haruki Musha, Moroe Beppu, Rie Karasawa, Kazuo Yudoh

**Affiliations:** 1 Department of Sports Medicine, St. Marianna University School of Medicine, 2-16-1 Sugao, Miyamae-ku, Kawasaki, Kanagawa 216-8511, Japan; E-Mails: aburainisuke@marianna-u.ac.jp (N.Y.); h2yoshioka@marianna-u.ac.jp (H.Y.); fujiya-1487@marianna-u.ac.jp (H.F.); musha@marianna-u.ac.jp (H.M.); 2 Department of Orthopedic Surgery, St. Marianna University School of Medicine, 2-16-1 Sugao, Miyamae-ku, Kawasaki, Kanagawa 216-8511, Japan; E-Mail: mbortho7@marianna-u.ac.jp; 3 Department of Frontier Medicine, Institute of Medical Science, St. Marianna University School of Medicine, 2-16-1 Sugao, Miyamae-ku, Kawasaki, Kanagawa 216-8511, Japan; E-Mail: r2karasawa@marianna-u.ac.jp

**Keywords:** osteoarthritis, DNA repair enzyme, oxidative stress, chondrocytes, Apex 2 (apurinic/apyrimidinic endonuclease 2)

## Abstract

Apurinic/apyrimidinic endonuclease 2 (Apex 2) plays a critical role in DNA repair caused by oxidative damage in a variety of human somatic cells. We speculated that chondrocyte Apex 2 may protect against the catabolic process of articular cartilage in osteoarthritis (OA). Higher levels of Apex 2 expression were histologically observed in severely compared with mildly degenerated OA cartilage from STR/OrtCrlj mice, an experimental model which spontaneously develops OA. The immunopositivity of Apex 2 was significantly correlated with the degree of cartilage degeneration. Moreover, the OA-related catabolic factor interleukin-1β induced the expression of Apex 2 in chondrocytes, while Apex 2 silencing using small interfering RNA reduced chondrocyte activity *in vitro*. The expression of Apex 2 in chondrocytes therefore appears to be associated with the degeneration of articular cartilage and could be induced by an OA-related catabolic factor to protect against the catabolic process of articular cartilage. Our findings suggest that Apex 2 may have the potential to prevent the catabolic stress-mediated down-regulation of chondrocyte activity in OA.

## 1. Introduction

Osteoarthritis (OA) symptoms and joint destruction become aggravated both as the disease advances and with aging, because damage progresses over time and articular cartilage has a reduced capacity for self-repair. Although it is therefore necessary to establish preventive measures and early treatment strategies against articular cartilage degeneration, effective therapy for the early stages of OA is still being developed. Clarification of the exact pathogenesis of cartilage degeneration and new therapeutic strategies for OA are urgently needed. 

Numerous reports have demonstrated that chondrocytes produce excess amounts of reactive oxygen species (ROS) in the form of superoxide, nitric oxide, hydrogen peroxide, and peroxynitrite [1,2,3,4], as well as proinflammatory cytokines and chemokines in response to the mechanical forces imposed on articular cartilage [5,6,7]. This results in the depolymerization of hyaluronic acid and chondrocyte death in OA. The resulting ROS mediates various cellular signaling pathways [8], but higher levels of ROS can induce oxidative DNA damage leading to cellular apoptosis that contributes to aging, and malignant and degenerative diseases including OA. There is a general consensus that the degeneration of articular cartilage is partially mediated by oxygen free radicals [9,10]. However, the exact mechanism of ROS-mediated articular cartilage degeneration remains unclear. Moreover, the status of cellular antioxidants in OA chondrocytes is also uncertain.

Oxidative DNA lesions accumulate in nuclear and mitochondrial genomes during aging, and this can increase dramatically in age-related neurodegenerative diseases such as Parkinson’s disease [11,12,13] and Alzheimer’s disease [14,15,16,17]. We previously reported that oxidative DNA damage in chondrocytes accumulates in the degenerated articular cartilage in OA, reducing its maintenance potential [4]. Our *in vitro* study also indicated that the OA-related catabolic factors interleukin (IL)-1β and hydrogen peroxide (H_2_O_2_) induce the down-regulation of chondrocyte activity [18] as well as the overexpression of oxidized forms of nucleic acids, guanine (8-oxoguanine), and tyrosine (nitrotyrosine) in chondrocytes.

Oxidative damage to nucleic acids is counteracted by the activity of several enzymes such as apurinic/apyrimidinic (AP) endonuclease 2 (Apex 2). It has been reported that AP sites commonly occur in DNA molecules following spontaneous hydrolysis, DNA damaging agents, or DNA glycosylases that remove specific abnormal bases [19]. AP expression sites are pre-mutagenic lesions that can prevent normal DNA replication from occurring, so cellular mechanisms identify and repair such sites. More recently, it was revealed that Apex 2 is involved in the critically important DNA repair pathway [20]. The base excision repair pathway is largely responsible for the repair of oxidative stress-induced DNA damage, but the molecular mechanism of the DNA damage checkpoint activation had not been elucidated. However, Willis *et al*. [21] have now provided evidence for the trigger of checkpoint kinase phosphorylation by oxidative stress. They showed that Apex 2 was required for the generation of replication protein and the recruitment of a checkpoint protein complex to DNA damage sites.

We postulated that the change of cellular anti-oxidative activity that occurs through the Apex 2-associated DNA repair pathway in degenerated articular cartilage may, at least in part, participate in OA cartilage degeneration. In the present study, we found that Apex 2 expression in chondrocytes was induced by OA-related catabolic factors *in vitro*, and was associated with the degeneration of articular cartilage in an *in vivo* OA mouse model. Furthermore, Apex 2 silencing using small interfering (si)RNA reduced chondrocyte activity *in vitro*. These findings suggest that Apex 2 may help prevent the catabolic stress-induced down-regulation of chondrocyte activity in OA. Our current study therefore suggests a novel mechanism for oxidative stress-mediated DNA damage in OA.

## 2. Results and Discussion

### 2.1. Immunopositivity of Apex 2 (Apurinic/Apyrimidinic Endonuclease 2) Protein in Degenerated Articular Cartilage of Spontaneous OA (Osteoarthritis) Mice

Apex 2 immunopositivity was compared between degenerated and non-degenerated articular cartilage sections in an OA mouse model to understand the relationship between oxidative damage and OA development. In the present study, we used OA model mice, STR/OrtCrlj mice, which spontaneously develop an osteoarthritic process [22]. The STR/ort strain of mouse is an acceptable animal model for studying the development of OA. About 85% of all male mice naturally develop OA in the medial tibial plateau. A number of biochemical features such as matrix proteoglycan depletion by matrix metalloproteinase and aggrecanase are also similar to changes found in human osteoarthritic cartilage. In contrast, spontaneous OA in STR/ort mice seems to be unlikely due to greater vulnerability to mechanical stress. Poulet B. *et al*. [23] indicated that genetic susceptibility to OA in STR/ort mice was not necessarily linked to a greater vulnerability of articular cartilage to mechanical damage. It is unlikely that an animal model would express all of the features observed in the human OA. Although relative contributions of genetic and mechanical factors to OA still remain unclear, the STR/ort model provides a unique opportunity to investigate the events involved in the initiation of OA and its progression.

OA is characterized by cartilage degeneration, synovial fibrosis, and bone remodeling (osteophyte formation). The osteophyte formation is considered to be a response to the altered joint loading and the changed metabolic microenvironment in the joint. Recent report demonstrated that both transforming growth factor (TGF) and bone morphogenetic factor (BMP)-2 induced the osteophyte formation during the progression of OA [24]. In addition to the confirming the important role of TGF in OA development, it has been reported that transglutaminases-2 (TG2) differently influences cartilage destruction and bone remodeling (osteophyte) in surgical OA model mice following surgically-induced joint instability [25]. However, the role of these three factors, TGF, BMP-2 and TG2, in the cartilage degeneration and bone remodeling in STR/Ort mice (spontaneous OA model mice) remains unknown. Further studies are needed to understand the property and usefulness of STR/Ort mice.

**Figure 1:**
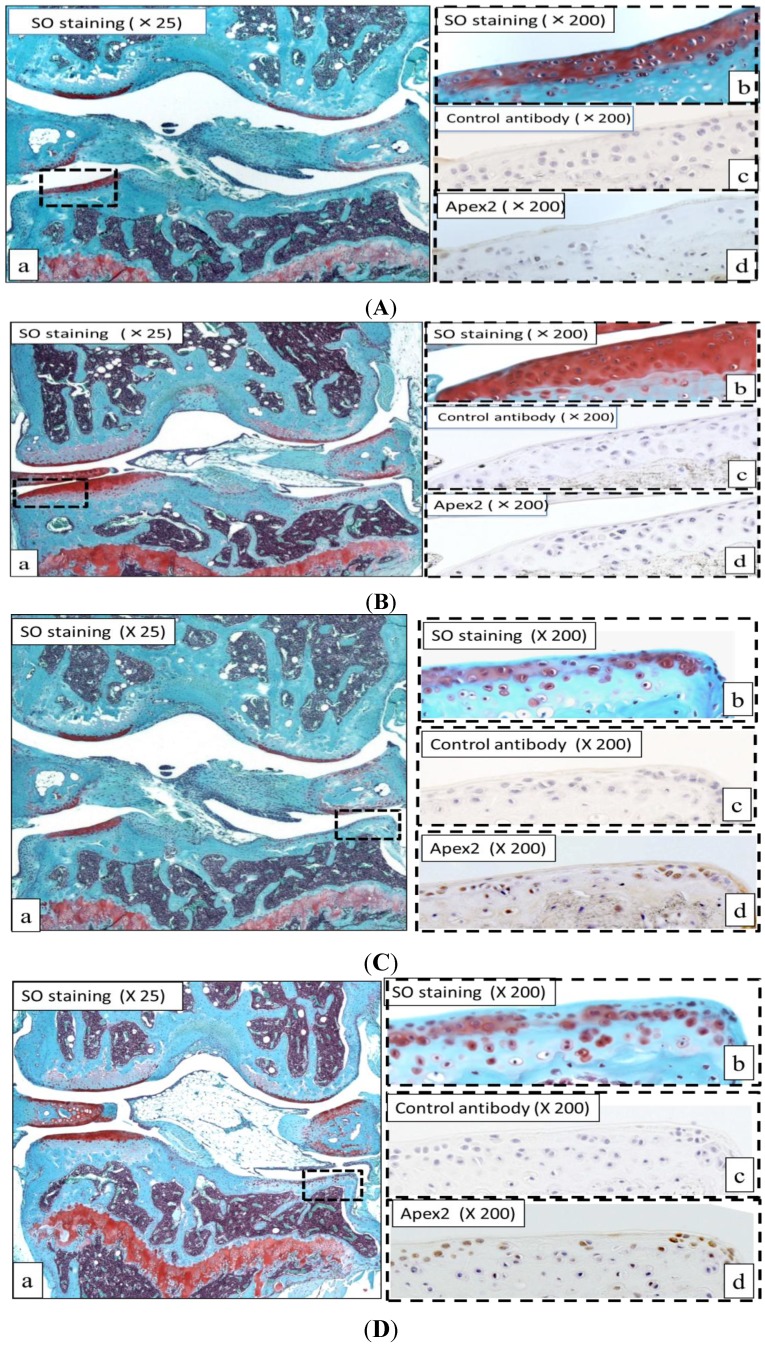
Expression of Safranin O (SO) staining and Apex 2 (apurinic/apyrimidinic endonuclease 2) immunohistological staining in articular cartilage of STR/OrtCrlj mice. (**A**,**B**) Representative images of Safranin O staining and Apex 2 immunohistological staining in mild osteoarthritis (OA) articular cartilages from STR/OrtCrlj mice; Safranin O staining (×25) (**a**);Safranin O staining (×200) (**b**); Control antibody (**c**);Apex2 immunostaining (**d**). Almost of the mildly degenerated cartilages were negative for Apex 2 immunostaining; (**C**,**D**) Representative images of Safranin O staining and Apex 2 immunohistological staining in severe OA articular cartilages from STR/OrtCrlj mice; Safranin O staining (×25) (**a**); Safranin O staining (×200) (**b**); Control antibody (**c**); Apex 2 immunostaining (**d**). A higher expression pattern of Apex 2 was observed in the severely degenerated cartilages. Immunopositivity for Apex 2 was predominant in the severe OA cartilages compared with the mild degenerated cartilages.

Figure 1 shows representative images of Safranin O staining and Apex 2 immunohistological staining in the articular cartilage of STR/OrtCrlj mice.

Severe degeneration of articular cartilage, as determined by reduced Safranin O staining, chondrocyte loss, surface irregularities, and superficial clefts, was observed (Figure 1C,D) and evaluated according to the macroscopic visual Collins’ scale and the histological Mankin scale [26,27].

Faint immunostaining of the Apex 2 protein was observed in normal articular cartilage (Figure 1A,B). Most mildly degenerated cartilage showed extremely low Apex 2 immunopositivity while this was much higher in the severely degenerated cartilage. Immunohistochemical analysis revealed that Apex 2 expression was detected in an average of 34% of chondrocytes in the severely degenerated articular cartilage (17 positive cells/50 total cells under high-power magnification) (Figure 1C,D). Immunostaining for Apex 2 was most apparent in the degenerated articular cartilage that showed histological changes consistent with OA.

**Figure 2:**
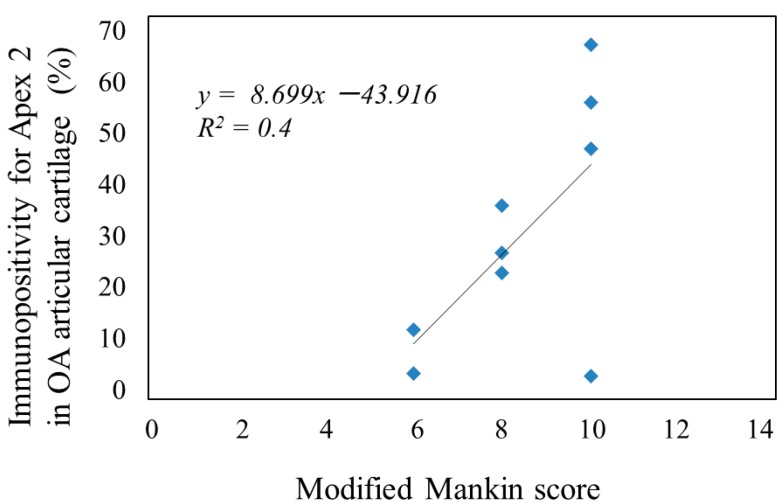
Correlation between Apex 2 expression and degree of cartilage degeneration in OA mouse model.

The mean number of Apex 2-positive chondrocytes in the articular cartilage correlated with the degree of cartilage degeneration determined by the modified Mankin score in mice (Figure 2). These findings clearly indicate that Apex 2 is expressed in chondrocytes within degenerated articular cartilage, but not in normal cartilage.

### 2.2. Expression of Apex 2 Protein in OA Chondrocytes

**Figure 3:**
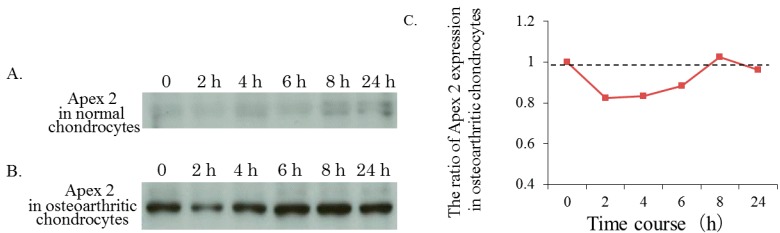
Expressions of Apex 2 in chondrocytes. (**A**) Expression of Apex 2 in normal chondrocytes from patients with traumatic femoral neck fractures in the presence of IL-1β (10 ng/mL); (**B**) Expression of Apex 2 in OA chondrocytes from patients with OA in the presence of IL-1β (10 ng/mL); and (**C**) Time course of Apex 2 expression in OA chondrocytes in the presence of IL-1β (10 ng/mL). The level of Apex 2 expression was analyzed as a relative ratio against the level of expression of Apex 2 at initial phase (0 hour-incubation).

Normal chondrocytes from non-degenerated articular cartilage from patients with traumatic femoral neck fractures did not express Apex 2 protein (Figure 3A). Even in the presence of the OA-related catabolic factor IL-1β (10 ng/mL), the expression of Apex 2 in normal chondrocytes was barely detectable at all time-points studied (Figure 3A).

By contrast, OA chondrocytes spontaneously expressed higher levels of Apex 2 protein (Figure 3B).

In those OA chondrocytes treated with IL-1β, the expression of Apex 2 protein temporarily decreased during the first 2 h of incubation but then gradually increased and recovered (Figure 3C).

These findings suggest that the expression of Apex 2 in chondrocytes is involved in the pathogenesis of OA. Interestingly, the OA-related catabolic factor IL-1β induced the expression of Apex 2 in chondrocytes, indicating that this may also participate in cartilage degeneration.

### 2.3. siRNA-Mediated Downregulation of APEX2 Reduces Chondrocyte Activity

**Figure 4:**
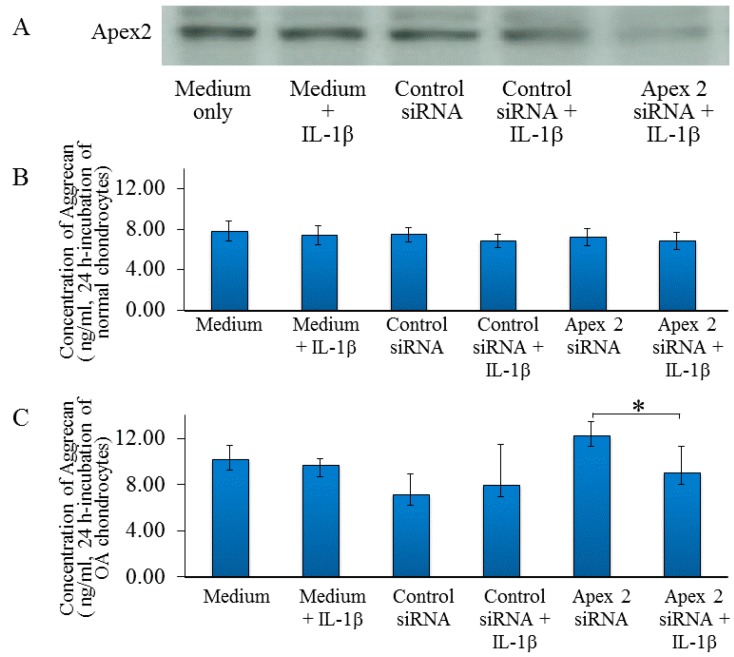
siRNA-mediated downregulation of Apex 2 mRNA reduces chondrocyte activity. To address whether Apex 2 is necessary to prevent catabolic factor-induced changes in chondrocyte activitys, the Apex 2 mRNA levels were silenced using siRNAs. (**A**) The siRNA against Apex 2 reduced the expression of Apex 2 protein expression by ~40% compared with scrambled control siRNA treatment; (**B**) In normal chondrocytes, silencing of Apex 2 with siRNAs did not influence the chondrocyte activity evaluated by aggrecan production;(**C**) In OA chondrocytes, silencing of Apex 2 with siRNAs significantly inhibited the chondrocyte activity evaluated by aggrecan production (* *p* < 0.05).

To determine whether Apex 2 is necessary to prevent catabolic factor-induced changes in chondrocyte activity, Apex 2 was silenced using siRNA. Compared with scrambled control siRNA treatment, the siRNA against Apex 2 reduced Apex 2 protein expression by 59%~81% as shown by western blotting (Figure 4A). There was no significant difference in the production of proteoglycan from normal chondrocytes between controls and Apex 2 siRNA-treated groups even in the presence or absence of IL-1β (Figure 4B). Notably, Apex 2 silencing reduced OA chondrocyte activity by 75% as evaluated by proteoglycan production (Figure 4C). These findings suggest that Apex 2 siRNA silencing reduced the production of proteoglycan from OA chondrocytes. Because proteoglycan is a major component of articular cartilage, our results indicate the importance of Apex 2 in chondrocyte activity and the maintenance of articular cartilage. Thus, the downregulation of Apex 2 in chondrocytes appears to be implicated in the development of OA.

Although ROS mediate various cellular signaling pathways, higher levels of ROS can induce oxidative DNA damage and the resultant cellular death contributes to malignancy and degenerative diseases. In the present study, we investigated differences in expression of antioxidant error-avoiding mechanisms between normal articular cartilage and OA degenerated cartilage using a specific antibody against Apex 2. The major finding of our study is the accumulation of Apex 2 in OA chondrocytes within the degenerated articular cartilage of OA model mice, suggesting that oxidative DNA damage and depletion of cellular antioxidants are involved in the pathogenesis of OA. The observed Apex 2 immunoreactivity in degenerated cartilage is consistent with previous reports of other diseases. Our results indicate that oxidative damage accumulates in OA degenerated articular cartilage and that Apex 2 does not repair the damage efficiently, which may lead to downregulation of chondrocyte activity and the maintenance potential of OA articular cartilage.

We detected significant changes between control and Apex 2-deficient chondrocytes in terms of proteoglycan production. Our data support a role for Apex 2 in preventing stress-induced chondrocyte dysfunction in human chondrocytes that might be associated with the maintenance of articular cartilage. This extends the work of others by revealing that oxidative stress is involved in the pathogenesis of OA.

Our result indicate that osteoarthritic chondrocytes, but not normal chondrocytes, express Apex 2 and that Apex 2 may, at least in part, participate in the maintenance of chondrocyte activity in OA. Indeed, many reports clearly demonstrated that mechanical force to articular cartilage induced the excess amount of ROS from chondrocytes and that mechanical stress-induce ROS as an oxidative stress damaged chondrocytes and cartilage matrix. We have already observed the overexpression of oxidized forms of nucleic acids, guanine (8-oxoguanine), and tyrosine (nitrotyrosine) in osteoarthritic chondrocytes, but not normal chondrocyte [4].

Regarding other diseases, DNA repair enzymes are already known to be expressed in neurodegenerative diseases (Parkinson’s disease [11,12,13], Alzheimer’s disease [14,15,16,17]). In the present study, we have found that osteoarthritic chondrocytes from degenerated cartilage expressed a higher level of Apex 2 (Figure 3B). In contrast to OA chondrocytes, normal chondrocytes from normal articular cartilage did not express the Apex 2 (Figure 3A). From these results, we would like to conclude that, in OA condition, chondrocytes in degenerated articular cartilage ubiquitously express Apex 2 in response to OA-associated factors and/or damages. In our opinion, Apex 2 expression may be needed to protect against catabolic stresses, involving the oxidative stress, and against further down-regulation of chondrocyte activity in OA. Thus, we think that marginal change of Apex 2 expression in osteoarthritic chondrocytes may affect chondrocyte activity. Indeed, Apex 2 silencing reduced OA chondrocyte activity (as evaluated by proteoglycan production) in the presence of catabolic factor IL-1β. Although further studies are need to clarify the exact mechanism and involvement of Apex 2 expression in OA chondrocytes, we would like to conclude that expression of Apex 2 may be implicated with pathogenesis of OA.

## 3. Experimental Section

### 3.1. Chondrocyte Isolation from Human Articular Cartilage

Human articular cartilage samples were obtained from knee joints during arthroplastic knee surgery for OA (*n* = 7; mean age, 76.1 years (range, 64–88 years)) and non-degenerated articular cartilage from patients with traumatic femoral neck fracture (*n* = 4; mean age, 78.8 years (range, 72–83 years)) after obtaining informed consent from patients. All samples were obtained with the informed consent from the patients, and the study protocol was approved by the ethics committee of St. Marianna University.

Human cartilage explants were cut into small pieces, washed with phosphate-buffered saline (PBS) and digested with 1.5 mg/mL collagenase B (Sigma, St. Louis, MO, USA) in Dulbecco’s modified Eagle’s medium (DMEM) (Sigma) at 37 °C overnight on a shaking platform. The isolated chondrocytes were centrifuged, washed three times with PBS, resuspended and cultured in DMEM supplemented with 10% heat-inactivated fetal calf serum, 2 mM l-glutamine, 25 mM HEPES (2-[4-(2-hydroxyethyl)-1-piperazinyl] ethanesulfonic acid), and 100 U/mL penicillin and streptomycin at 37 °C in a humidified atmosphere of 95% air and 5% CO_2_.

### 3.2. OA Model Mice

The study was performed on STR/OrtCrlj mice, an experimental model which spontaneously develops an osteoarthritic process. Male STR/OrtCrlj mice (12 weeks of age) were purchased from Charles River Japan (Yokohama, Japan). The mice were maintained in a temperature (23–25 °C)-, humidity (40%–60%)-, and light-controlled environment with free access to a moderate fat diet (Japan SLC Co., Ltd., Shizuoka, Japan) and water. They were acclimatized for at least 1 week before the start of the study.

To clarify the involvement of Apex 2 in the progression of cartilage degeneration, we investigated its expression levels in chondrocytes prior to the development of cartilage degeneration in the OA mouse model. Mice were euthanized by administration of diethyl ether at 4, 8, 12, 16, 20, or 24 weeks (*n* = 4 per time point) after the operation. After harvesting of both knees, the articular bone and cartilage were obtained and paraffin blocks were prepared using standard histologic procedures. STR/OrtCrlj mouse articular cartilage samples with subchondral bone were fixed for 2 days in 4% paraformaldehyde solution and then decalcified in 4% paraformaldehyde containing 0.85% sodium chloride and 10% acetic acid. Subsequently, the tissues were dehydrated in a series of ethanol solutions, infiltrated with xylene, embedded in paraffin and cut into 6-μm sections.

### 3.3. Immunohistochemistry for Apex 2 in Mouse OA Articular Cartilage

For histological examination of the degree of articular cartilage degeneration, some sections were stained with Safranin O-fast green to determine the loss of proteoglycans. Each cartilage sample was evaluated histologically and macroscopically according to the scales of Mankin and Collins (Table 1) [26,27]. The mean damage score for each animal was used to determine the mean ± standard deviations for each group. Three independent observers assessed cartilage damage in a blinded manner.

**Table 1:** Modified Mankin Score (criteria for histological evaluation).

Category	Score	Criteria for Histologic Evaluation
Safranin O-Fast Green Staining	0	uniform staining throughout articular cartilage
	1	loss of staining in the superficial zone for less than one-half of the length of the plateau
	2	loss of staining in the superficial zone for one-half or more of the length of the plateau
	3	loss of staining in the superficial and middle zones for less than one-half of the length of the plateau
	4	loss of staining in the superficial and middle zones for one-half or more of the length of the plateau
	5	loss of staining in all 3 zones for less than one-half of the length of the plateau
	6	of staining in all 3 zones for one-half or more of the length of the plateau
Chondrocyte Loss	0	no decrease in cells
	1	minimal decrease in cells
	2	moderate decrease in cells
	3	marked decrease in cells
	4	very extensive decrease in cells
Structure	0	normal
	1	surface irregularities
	2	1–3 superficial clefts
	3	3 superficial clefts
	4	1–3 clefts extending into the middle zone
	5	>3 clefts extending into the middle zone
	6	1–3 clefts extending into the deep zone
	7	>3 clefts extending into the deep zone
	8	clefts extending to calcified cartilage

Serial sections of paraffin-embedded bone and cartilage tissues were cut and immunostained with antibodies against Apex 2. These sections were treated with 3% H_2_O_2_, followed by blocking of nonspecific protein binding with a blocking agent (Protein Block, Dako, Carpinteria, CA, USA). The sections were then incubated with monoclonal antibodies against Apex 2 (1:400 dilution; Abcam Inc., Cambridge, UK) for 1 h at room temperature, followed by incubation with biotinylated goat anti-mouse IgG (Dako) for 30 min at room temperature. After washing with PBS, the sections were incubated with a streptavidin-horseradish peroxidase complex (LSAB2 Kit; Dako) for 10 min at room temperature. Diaminobenzidine (Sigma) was used as a visible peroxidase reaction product, and the sections were counterstained with Mayer’s hematoxylin (Sigma). The number of Apex 2-positive cells was counted in five degenerated articular cartilage areas under high-power magnification (×400) for each case, and the mean number of positive cells per high-power field was calculated.

### 3.4. Effects of OA-Related Catabolic Factors on Apex 2 Expression in Chondrocytes

To examine the effects of an OA-related catabolic factor, IL-1β, on Apex 2 expression, chondrocytes were incubated in the presence or absence of IL-1β (10 ng/mL) for 24 h at 37 °C in a humidified atmosphere of 95% air and 5% CO_2_. After harvesting of the cultured cells, protein samples were collected for enzyme-linked immunosorbent assay (ELISA) and immunoblotting analyses.

The expression levels of Apex 2 in chondrocytes were analyzed by western blotting. The antibodies used included polyclonal antibodies against Apex 2 (Abgent, San Diego, CA, USA) and the corresponding secondary antibody conjugated with horseradish peroxidase (DAKO). The antibody-bound protein bands were visualized by X-ray films (Fuji Photo Film Co., Ltd., Tokyo, Japan). Densitometry of the signal bands was determined using ImageJ software [28].

### 3.5. Transient Apex 2 siRNA Transfection and Western Blotting

Stealth RNAi negative control duplexes that silence Apex 2 and scrambled siRNA controls were purchased from Life Technologies (Carlsbad, CA, USA). All experiments were performed with sense (GCCAUGUGAUCAUUCUGGGUGACCU) and antisense (AGGUCACCCAGAAUGAUCACAUGGC) siRNAs. Briefly, chondrocytes were seeded at 8 × 10^4^ cells/6-well plate 24 h before transfection, which was carried out using 40 μL of Lipofectamine 2000 (Life Technologies: Carlsbad, CA, USA), and 100 pmol of siRNA. Cells were harvested by trypsinization after 24 h incubation for protein isolation and determination of the protein concentration, before use in subsequent experiments to clarify the effects of Apex 2 expression on chondrocyte activity.

To determine the efficacy of transient Apex 2 siRNA transfection, the expression levels of Apex 2 protein in chondrocytes were analyzed by western blotting as described above.

### 3.6. Effects of Apex 2 Expression on Chondrocyte Activities

To clarify the effects of Apex 2 on chondrocyte activity, we examined the production levels of the cartilage matrix component proteoglycans in Apex 2 siRNA-treated chondrocytes and control chondrocytes *in vitro*. Apex 2 siRNA-treated chondrocytes, and positive and negative control chondrocytes were incubated in the presence or absence of IL-1β (10 ng/mL) for 24 h at 37 °C in a humidified atmosphere of 95% air and 5% CO_2_. Subsequently, the conditioned culture medium was collected and stored at −80 °C until required for analysis.

To examine the effects of Apex 2 on chondrocyte anabolic activity, the levels of proteoglycan production by cultured chondrocytes were measured using an ELISA kit (PG EASIA; BioSource Europe S.A., now part of Life Technologies Corporation, Carlsbad, CA, USA) in accordance with the manufacturer’s protocol.

### 3.7. Statistical Analysis

The results for each experimental condition were determined from the mean of triplicate trials. Data were expressed as means ± standard deviation. A two-tailed Student’s *t*-test was used to assess the significance of differences between two groups. Analysis of variance was used for comparisons of more than two groups, and differences between two groups within the set were analyzed by a Fisher’s protected least-significant difference test. Probability values of <0.05 were considered significant.

## 4. Conclusions

In conclusion, our data establish an innovative role for Apex 2 in protecting articular cartilage and chondrocytes against catabolic stress-induced chondrocyte dysfunction in OA.

## References

[1] Evans C.H., Stefanovic-Racic M. (1996). Nitric oxide in arthritis. Method.

[2] Abramson S.B., Attur M., Amin A.R., Clancy R. (2001). Nitric oxide and inflammatory mediators in the perpetuation of osteoarthritis. Curr. Rheumatol. Rep..

[3] Del Carlo M., Looser R.F. (2002). Nitric oxide-mediated chondrocyte cell death requires the generation of additional reactive oxygen spesies. Arthritis Rheumatol..

[4] Yudoh K., Nguyen T., Nakamura H., Hongo-Masuko K., Kato T., Nishioka K. (2005). Potential involvement of oxidative stress in cartilage senescence and development of osteoarthritis: Oxidative stress induces chondrocyte telomere instability and downregulation of chondrocyte function. Arthritis Res. Ther..

[5] Agarwal S., Deschner J., Long P., Verma A., Hofman C., Evans C.H., Piesco N.P. (2004). Role of NF-κB transcription factors in anti-inflammatory and pro-inflammatory actions of mechanical signals. Arthritis Rheumatol..

[6] Guilak F., Formor B., Keefe F.J., Kraus V.B., Olson S.A., Pisetsky D.S., Setton L.A., Weinberg J.B. (2004). The role of biomechanics and inflammation in cartilage injury and repair. Clin. Orthop. Relat. Res..

[7] Budo K., Angelica K.L., Jacob F., Thomas P., Alan J.G., Michael S. (2005). Pathomecanism of cartilage destruction by mechanical injury. Ann. Anat..

[8] Henrotin Y.E., Bruckner P., Pujol J.P. (2003). The role of reactive oxygen species in homeostasis and degradation of cartilage. Osteoarthr. Cartil..

[9] Antonia F.C., Catrin M.D., Ming D.L., Beverley F. (2008). Oxidative DNA damage in osteoarthritic porcine articular cartilage. J. Cell Physiol..

[10] Valery A., Romuald C., Dragoslav M., Pascal C., Abderrahim L. (2007). Ractive oxygen species and superoxide dismutases: Role in joint disease. Jt. Bone Spine.

[11] Michelle B.M., Lucas S.S., Bennett V.H. (2010). Mitochondrial dysfunction in neurodegenerative disease and cancer. Environ. Mol. Mutagen..

[12] Maria M., David M.L., Lorena M.B., Joaquin A., Miguel A.M., Criatina U. (2012). Mitochondrial respiratory chain dysfunction: Implications in neurodegeration. Free Radic. Biol. Med..

[13] Zuo L., Motherwell M.S. (2013). The impact of reactive oxygen species and genetic mitochondrial mutations in parkinson’s disease. Gene.

[14] Xiongwei Z., Hyoung-gon L., George P., Mark A.S. (2007). Alzheimer disease, the two-hit hypothesis: An update. Biochim. Biophys. Acta.

[15] Sompol P., Ittarat W., Tangpong J., Chen Y., Doubinskaia I., Batinic-Haberle I., Abdul H.M., Buttrefield D.A., Clair D.K.S.T. (2008). A neuronal model of Arzheimer’s disease: An insight into the mechanisms of oxidative stress- mediated mitochondrial injury. Neuroscience.

[16] Daniel G., Rafal R., Agnieszka S., Tomasz D., Krzysztof N., Maciej K., Aleksander A., Ryszard O. (2008). Oxidative stress and Oxidative DNA damage is characteristic for mixed Alzheimer diseade/vasucular dementia. J. Neurol. Sci..

[17] Russell H.S. (2011). Brain aging, Alzheimer’s disease, and mitochondria. Biochim. Biophys. Acta.

[18] Davis C.M., Guilak F., Weinberg J.B., Fermor B. (2008). Reactive nitrogen and oxygen spesies in interleukin-1-mediated DNA damage associated with osteoarthritis. Osteoarthr. Cartil..

[19] Georgiadis M.M., Luo M., Gaur R.K., Delaplane S., Li X., Kelley M.R. (2008). Evolution of the redox function in mammalian apurinic/apyrimidinic endonuclease. Mutat. Res..

[20] Fishel M.L., Kelley M.R. (2007). The DNA base excision repair protein Ape1/Ref-1 as a therapeutic and chemopreventive target. Mol. Aspects Med..

[21] Willis J., Patel Y., Lentz B.L., Yan S. (2013). APE2 is required for ATR-Chk1 checkpoint activation in response to oxidative stress. Proc. Natl. Acad. Sci. USA.

[22] Das-Gupta E.P., Lyons T.J., Hoyland J.A., Lawton D.M., Freemont A.J. (1993). New histological observations in spontaneously developing osteoarthritis in the STR/ORT mouse questioning its acceptability as a model of human osteoarthritis. Int. J. Exp. Pathol..

[23] Poulet B., Westerhof T.A.T., Hamilton R.W., Shefelbine S.J., Pitsillides A.A. (2013). Spontaneous osteoarthritis in Str/ort mice is unlikely due to greater vulnerability to mechanical trauma. Osteoarthr. Cartil..

[24] Blaney Davidson E.N., Vitters E.L., van Beuningen H.M., van de Loo F.A.J., van den Berg W.B., van der Kraan P.M. (2007). Resemblance of osteophytes in experimental osteoarthritis to transforming growth factor beta-induced osteophytes: Limited role of bone morphogenetic protein in early osteoarthritic osteophyte formation. Arthritis Rheumatol..

[25] Orlandi A., Oliva F., Taurisano G., Candi E., di Lascio A., Melino G., Spagnoli L.G., Tarantino U. (2009). Transglutaminase-2 differently regulates cartilage destruction and osteophyte formation in a surgical model of osteoarthritis. Amino Acids.

[26] Mankin H.J., Dorfman H., Lippiello L., Zarins A. (1971). Biochemical and metabolic abnormalities in articular cartilage from osteoarthritic human hips. II. Correlation of morphology with biochemical and metabolic data. J. Bone Jt. Surg. Am..

[27] Furman B.D., Strand J., Hembree W.C., Ward B.D., Guilak F., Olson S.A. (2007). Joint degeneration following closed intraarticular fracture in the mouse knee: A model of posttraumatic arthritis. J. Orthop. Res..

[28] Schneider C.A., Rasband W.S., Eliceiri K.W. (2012). NIH Image to ImageJ: 25 years of image analysis. Nat. Methods.

